# The Optical Society

**Published:** 1904-05-14

**Authors:** W. Salt

**Affiliations:** hon secretary of the Optical Society


### EDITOR'S LETTER-BOX.

### THE OPTICAL SOCIETY.

*To the Editor of* "The Hospital."

Mr. W. Salt, hon secretary of the Optical Society, writes: With reference to a recent article in the *Times* newspaper, under the heading "Testing the Eyesight of the Nation," which is commented upon in your issue of April 16th, I am authorised by my council to state that the Optical Society, which is mainly a scientific society as I show in my subsequent quotation of its objects, does not at this stage commit itself to any definite opinion on the sight-testing question, nor can it accept responsibility for the views of individual members; moreover it does not organise or conduct examinations, or grant "diplomas" of any sort whatever.

In the article I quote it is mentioned that the *Optician and Photographic Trades Review* is stated to be the official organ of the Optical Society. That journal is the official medium for the publication of the Society's notices and proceedings, but it is in no sense authorised to voice independently the opinions of the Optical Society as a body. Moreover, I have the authority of the editor of the *Optician and Photographic Trades Review* for the assertion that neither he nor the *Official Journal* had anything to do with sending out the so-called "circular communication."

As a matter of interest to your readers, I shall be glad if you will permit me to state that the objects of the Optical Society (which was founded in the year 1899) are:—

(*a*) To promote intercourse between those interested in the science of optics in the United Kingdom, the colonies, or foreign countries.

(*b*) To foster and disseminate knowledge of optical matters among its members.

(*c*) To arrange lectures, discussions, and demonstrations on optical science, and optical instruments.

(*d*) To provide its members with information on all optical subjects of interest.

(*e*) To promote the establishment, adoption, and recognition of standard sizes, gauges, calibres, templets, and methods for universal use with all optical and other scientific instruments.

(*f*) To print, publish, sell, lend, or distribute the proceedings or reports of the Society, or any papers or communications on optical science or subjects connected therewith.

[.·. We may as well point out that the article on "'Opticians' and Sight Testing," which appeared in our issue of April 16th, contains no mention of the "Optical Society" or of the *Optician and Photographic Trades Review*. It deals only with the broad general issue of whether, in the interests of the public, it is right or wrong to allow anything resembling official guarantee of capacity to be obtainable by individuals who have not that knowledge of human anatomy, physiology, and general medicine, which is necessary for the safe and proper management of cases of defective vision.—Ed. Hospital.]

